# Molecular identification and diversity assessment of Tyrrhenian *Romulea* species (Iridaceae)

**DOI:** 10.1371/journal.pone.0358245

**Published:** 2026-09-15

**Authors:** Alex Baumel, Virgile Noble, Cyllène Chatellier, Juan Viruel, Stephen Mifsud, Henri Michaud, Alain Delage, Lisandru Leandri, Andrea Lallai, Gianluca Iiriti, Gianniantonio Domina, Gabriele Casazza, Pere Fraga-Arguimbau, Baptiste Pierre, Anouar Toumi, Frédéric Médail

**Affiliations:** 1 Aix Marseille Université, Avignon Université, CNRS, IRD, IMBE, Marseille, France; 2 Conservatoire Botanique National Méditerranéen, Hyères, France; 3 Escuela Politécnica Superior de Huesca. Universidad de Zaragoza, Huesca, Spain; 4 Instituto de Biocomputación y Física de Sistemas Complejos (BIFI), Universidad de Zaragoza, Zaragoza, Spain; 5 Royal Botanic Gardens, Kew, Richmond, United Kingdom; 6 EcoGozo Directorate, Ministry for Gozo and Planning, Victoria, Gozo, Malta; 7 Conservatoire Botanique National de Corse, Corte, France; 8 University of Cagliari, Cagliari, Italy; 9 Department of Agricultural, Food and Forest Sciences, University of Palermo, Palermo, Italy; 10 University of Genova, Genova, Italy; 11 Institut Menorquí d’Estudis, Maó, Minorca, Spain; 12 UMR AGAP Institute, Univ. Montpellier, CIRAD, INRAE, Institut Agro Montpellier, Montpellier, France; Griffith University, AUSTRALIA

## Abstract

Taxonomic assignments based only on morphology are often insufficient for delimiting species, particularly in complexes shaped by hybridization and polyploidy, where species boundaries are unclear. This limitation hinders progress in ecological, biogeographic and conservation research. The genus *Romulea*, distributed across Africa and the Mediterranean Basin, exemplifies this challenge. Despite its remarkable diversity, Mediterranean *Romulea* has not received much attention from genetic and molecular studies. Here, we present the first multilocus genotype analysis of Mediterranean *Romulea* taxa, focusing on the Tyrrhenian biogeographic province. Using target-capture sequencing with the universal Angiosperms353 kit, we generated genomic data for 272 individuals representing 18 putative taxa. Our findings reveal genetic groups that align with current taxonomy, the existence of cryptic divergence, and highlight the role of hybridization. Furthermore, analysis of intra-individual genetic diversity suggests one or several allopolyploid origins for Mediterranean *Romulea*. Four taxa (*R. assumptionis*, *R. revelieri*, *R. ligustica*, *R. rollii*) are consistently well differentiated across nuclear and plastid datasets, supporting their recognition as distinct species. In contrast, the widespread species *R. ramiflora* and *R. columnae* contain well-differentiated groups that may represent cryptic speciation. Several other taxa, including *R.* x *melitensis*, *R. corsica*, and *R. bulbocodium*, exhibit genomic signatures consistent with hybrid origins. Plastid and nuclear variation patterns are consistent with a hypothesis of rapid radiation in the Tyrrhenian region. These results provide a primary genomic framework for the integrative taxonomy of *Romulea*.

## Introduction

Taxonomic assignments constitute a critical foundation of biodiversity science, underpinning species description, classification, and biogeographical inference [1]. When based exclusively on morphology, they may introduce systematic biases that misinform evolutionary, ecological, and conservation studies. This limitation becomes particularly acute in plant species complexes distributed over wide geographical areas [2,3]. In such circumstances, robust species delimitation requires a quantitative assessment of genetic diversity structure. This approach is addressed through phylogeographic frameworks integrating population genetics and phylogenetics [4–6]. Despite their importance, species delimitation and the delineation of conservation units are rarely considered in Mediterranean plant species conservation literature [7–10].

In the Mediterranean Basin, several plant species complexes remain in urgent need of molecular systematics to disentangle taxonomic uncertainties and elucidate evolutionary relationships. The genus *Romulea* Maratti (Iridaceae) represents a paradigmatic case. This clade of geophytic monocots, distributed across Africa and the Mediterranean Basin, exhibits pronounced taxonomic richness and considerable morphological variation, and consequently a great taxonomic complexity. In the South African Cape Floristic Region, which constitutes the principal centre of diversity with approximately 76 recognized taxa, *Romulea* has been intensively characterized from a morphological, karyological, and floral biological standpoint [11]. In contrast, the Mediterranean *Romulea* species have received only partial treatment, often limited to early 20^th^-century monographs [12–14] or regional floristic accounts [15,16]. A significant discrepancy persists between the analytical approaches of the 20th century and the synthetic perspectives of works such as Flora Europaea [17], as seen in current international taxonomic checklists [18]. Recent detailed morphological assessment focused exclusively on three taxa of Malta archipelago [19], partly because taxon sampling in *Romulea* genus is challenging. Cytogenetic investigations [20] have revealed a predominance of tetraploid cytotypes (4x = 36 chromosomes), as well as pentaploid and hexaploid cytotypes for eleven Mediterranean taxa. This pattern contrasts sharply with the South African taxa, which are predominantly diploids although doubts subsist regarding the chromosome basic number [11]. Despite their diversity and originality, *Romulea* taxa remain severely underrepresented in molecular phylogenetic datasets, with a handful of DNA sequence accessions for studies in Systematics at family or order level [21–24]. Consequently, *Romulea*, especially in the Mediterranean, is a representative of the Linnean, Wallacean, and Darwinian shortfalls in biodiversity knowledge [25]: poor species delimitation and taxonomic confusion weaken distribution knowledge, which in turn undermines comprehensive sampling of variation.

The present study forms part of a broader integrative taxonomic initiative aimed at resolving species boundaries and reconstructing the evolutionary history of *Romulea* of the Mediterranean Basin. We concentrate here on the taxa occurring in an area centred around the Corsica-Sardinian microplate which separated from the continent 29 Myrs ago [26,27]. This region, called here Tyrrhenian area, exhibits high floristic diversity, an exceptional rate of endemism, and strong floristic links between the islands, highlighting a shared biogeographical history [28]. This is also the area where Mediterranean *Romulea* were mostly studied [15,16,19,20]. Our objectives are to (i) generate multilocus genetic data for a representative set of Mediterranean *Romulea* taxa, ii) delimit genetic groups corresponding to putative species-level lineages, iii) assess their evolutionary distinctiveness through population genetic and phylogenetic inference, and iv) reconcile molecular groupings with independent morphological identifications conducted by expert botanists.

## Materials and methods

### Sampling design

**Fig 1:**
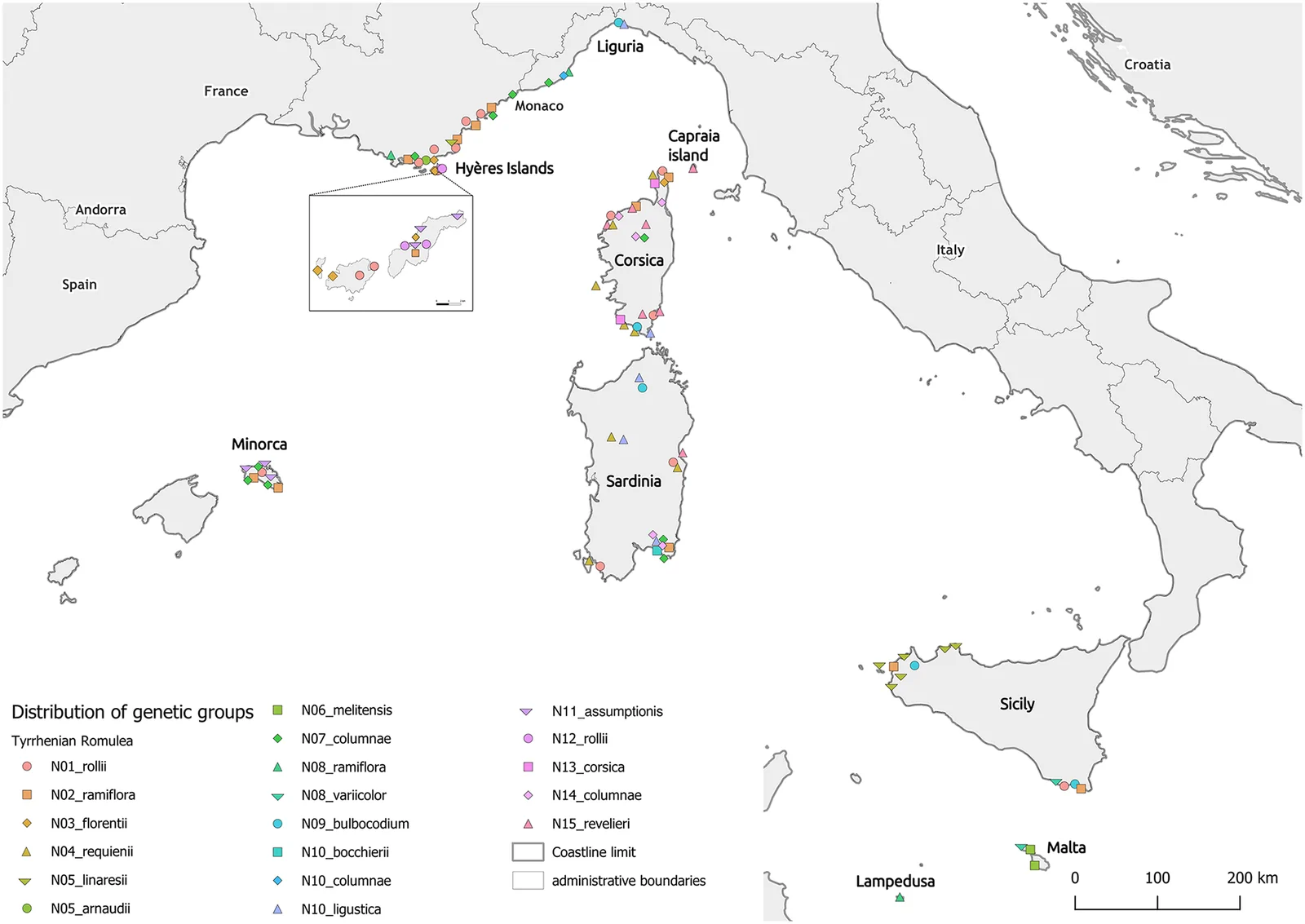
Sampling of *Romulea* populations. Symbols and colours according to the 19 genetic groups revealed by this study on the basis of multi-locus nuclear data. The map was built in with open source data [32].

Field collections of silica-dried leaves were conducted across four Mediterranean countries within the Tyrrhenian biogeographic province (Fig 1), targeting all taxa of *Romulea* occurring in this area (Table 1). Field work was performed by local botanists, represented locally by at least one author, and involved in plant conservation of their area with the appropriate permission when required. Only leaves were taken to avoid any local destruction. Whenever possible, a minimum of three populations per taxon and three individuals per populations were sampled to capture intra-population and inter-population genetic variation as recommended by [29]. Field identification relied on current taxonomic keys, with provisional assignments recorded *in situ* that was discussed during remote meetings. In cases where individuals of several species occurred in sympatry, potential hybrids were sought and identified by detecting atypical individuals exhibiting phenotypic traits intermediate between those of the putative parental species. Particular attention was paid to characters considered diagnostic in standard floras. As some hybrids in *Romulea* are known to favour vegetative reproduction [19], the occurrence of dense clusters of flowering shoots was also considered an additional indication of possible hybridization. Putative hybrids were recorded as such in Table 1. *Romulea rosea*, an alien species introduced from South Africa and naturalized in France, was also included. Additionally, DNA of *R. saldanhensis*, *R. dichotoma*, and *R. pratensis*, all native to South Africa, was obtained from the Royal Botanic Gardens, Kew DNA Bank, while sequence data for *R. monadelpha*, also from South Africa were obtained from PAFTOL [30]. Samples from South Africa were included as outgroups in the phylogenetic analysis of plastid data (see below). All metadata, including precise geographic coordinates and ENA [31] accession numbers (ENA project PRJEB85456) are provided in supporting information (S1 File).

**Table 1:** *Romulea* sampling. POWO = current taxonomy according to [18]; N = number of samples genotyped for this study. Chromosomes numbers (2n) are from [11,20]. Full metadata in Supporting information (S1 File).

Romulea taxon	POWO	N	Areas	2n
*R. arnaudii* Moret	Accepted	10	Provence	–
*R. arnaudii* x *columnae*		3	Provence	–
*R. arnaudii* x *rollii*		1	Provence	–
*R. assumptionis* Font Quer	*Romulea columnae* subsp. *assumptionis*	8	Minorca, Hyères Islands	56
*R. bocchierii* Frignani & Iiriti	Accepted	3	Sardinia	45
*R. bulbocodium* (L.) Sebast. & Mauri	Accepted	13	Liguria, Sardinia, Sicily	36
*R. columnae* Sebast. & Mauri	Accepted	37	Corsica, Liguria, Malta, Minorca, Provence, Sardinia	27, 45
*R. columnae* x *ramiflora*		3	Provence	–
*R. corsica* Jord. & Fourr.	Accepted	6	Corsica	–
*R. florentii* Moret	Accepted	17	Hyères Islands, Provence	–
*R. insularis* Sommier	Accepted	2	Capraia Island (Tuscan Archipelago)	45
*R.* x *jordanii* Beg.	Accepted	1	Corsica	–
*R. ligustica* Parl.	Accepted	14	Corsica, Liguria, Sardinia	36
*R. linaresii* Parl.	Accepted	14	Sicily	54
*R.* x *melitensis* Bég.	Accepted	6	Malta	–
*R. aff. ramiflora*		4	Minorca	36
*R. ramiflora* Ten.	Accepted	34	Corsica, Liguria, Minorca, Provence, Sicily	36
*R. requienii* Parl.	Accepted	23	Corsica, Sardinia	36
*R. revelieri* Jord. & Fourr.	Accepted	19	Corsica, Sardinia	36
*R. rollii* Parl.	*Romulea columnae* subsp. *rollii*	36	Hyères Islands, Minorca, Provence, Sardinia	36
*R. variicolor* Mifsud	Accepted	18	Malta, Sicily	–
*R. dichotoma* (Thunb.) Baker	Accepted	1	South Africa	30
*R. pratensis* M.P.de Vos	Accepted	1	South Africa	44
*R. rosea* (L.) Eckl.	Accepted	2	Provence (exotic)	18
*R. saldanhensis* M.P.de Vos	Accepted	1	South Africa	24

Chromosome counts obtained from [11,20], for Mediterranean and South African taxa, are presented in Table 1. Preliminary genome size estimates by flow cytometry, conducted for this study, revealed only a narrow range of variation (approximately 3 pg/2C) which was not consistent with chromosome counts variation. Technical issues due to the choice of internal standard or the conservation of the tissues were suspected, as well as the possibility of a negative correlation between chromosome size and chromosome number [20] and/or dysploidy. As a consequence, flow cytometry analyses did not provide reliable information on ploidy levels and were not used here.

### DNA extraction and quality control

Leaf tissue samples were silicagel dried for several weeks prior to DNA extraction, which was conducted at at the Molecular and Cell Biology facility of IMBE (Marseille, France). Approximately 20–30 mg of dried tissue was fragmented into pieces < 5 mm and homogenized using a FastPrep-24 tissue grinder (MP Biomedicals) with three glass beads per tube at the lowest speed (4.5 m/s) for 45 s, repeated once. Following centrifugation, genomic DNA was extracted using the Nucleospin II Kit (Macherey-Nagel) with the following modification: lysis was performed with 600 µl of PL1 buffer for 1 h at 65°C. To minimize carryover of debris, 250 µl of lysate were carefully pipetted from the supernatant, leaving ~200 µL at the bottom of the tube. thereby improving 230/260 purity ratios. DNA was eluted in two sequential steps (60 µl and 30 µl) of PE buffer. DNA concentration and purity were assessed first using a Nanodrop One spectrophotometer (Thermo scientific) and subsequently validated with a Qubit HS dsDNA fluorometric assay (HS kit, Thermo Fisher Scientific). Samples yielding < 10 ng/µl or suboptimal purity ratios (<1.0 at A260/A230) were re-extracted when tissue material was available. High-quality DNA extracts were normalized to a concentration between 8 and 11 ng/µl and organized into 24-sample pools.

### Genomic library preparation, target capture and sequencing

Multi-locus genetic data were obtained using the hybseq method [33] with the Angiosperms353 probe kit [34]. Normalized DNA samples were shipped to the IGENseq service (ICM, CHU Pitié-Salpêtrière, Paris, France) for library preparation, target enrichment, and sequencing. A total of 150 ng of genomic DNA per sample were subjected to enzymatic fragmentation for 15 min to generate insert sizes ranging 200–450 bp. Libraries were prepared with the NEBNext Ultra II FS DNA Library Prep Kit (New England Biolabs), quantified with Qubit HS and SPARK fluorometry, and equimolarly pooled in groups of 24 for hybrid capture with the Angiosperms353 probe kit. Hybridization-based target enrichment was performed with the MyBaits v5 kit according to the manufacturer’s standard protocol, using 500 ng of pooled libraries as input. Post-capture amplification consisted of 15 PCR cycles using KAPA HiFi HotStart polymerase (Roche sequencing solutions), followed by purification with Ampure XP magnetic beads. Sequencing was carried out on an Illumina NextSeq 2000 platform with paired-end 150 bp reads, generating approximately 400 million reads across 192 samples.

### Bioinformatics pipeline and genotyping

Variant calling was constrained by the absence of a reference genome and the unknown ploidy levels of several taxa. Therefore, we first generated a custom reference from target-capture assemblies to facilitate variant calling while minimizing interference from paralogous loci. Raw reads were demultiplexed and quality-filtered using fastp [35], retaining reads with Phred scores ≥30. A representative set of 42 *Romulea* samples was selected to assemble target loci using HybPiper v2.16 [36] with the Mega353 target reference [37]. The selection aimed to represent each taxon in each area where it was sampled (one individual by area and by taxon). The samples with the highest sequencing effort were chosen. Supercontig (exons and introns) were extracted, and loci present in fewer than 20 samples or >2 paralog warnings were excluded. Multiple sequence alignments were performed using MAFFT with default parameters [38], trimmed with TrimAl [39] in automatic mode and a final manual curation was conducted in AliView [40] to remove spurious regions. Consensus sequences for each locus were generated using the EMBOSS [41] cons tool and concatenated into a custom reference used for read mapping.

Variant discovery was conducted using BWA-MEM [42] for alignment, duplicate removal with SAMBAMBA [43], and SNP calling with FreeBayes [44] under the following parameters: ploidy = 2, use-best-n-alleles = 4, min-mapping-quality = 30, min-base-quality = 20, min-coverage = 6, min-alternate-count = 3, no-population-priors, hwe-priors-off. Resulting VCF files were filtered using BCFtools [45] to retain only bi-allelic SNPs with minor allele frequency ≥0.01.

Genotype calling was performed with polyRAD [46], following best practices for polyploid genotyping [47–49] with the aim to obtain continuous genotype in which the probability of presence of each allele is recorded; thus a tetraploid, for biallelic SNP may have an alle with a probability of 0.25, 0.5, or 0.75, giving more resolution than a presence/absence coding for estimating genotypes distances. Potential ploidy levels were parameterized as tetraploid. The vcf file was imported in polyRAD with the function VCF2RADdata with default parameters. The *H*ind/*H*_E_ index [50] was used to assess whether the genotypes analysed here follow a diploid or a polysomic polyploid inheritance. It is independent of genotype calling. *H*ind/*H*_E_ = (k-1)/k (1-F) where k is the ploidy level and F the inbreeding coefficient. For diploid or autotetraploid genomes the *H*ind/*H*_E_ is expected to be close to 0.5 and 0.75 respectively, whereas hybrid or allopolyploid will have values close to one. By fixing F we can provide an assessment of the ploidy level; after excluding outlier loci having *H*ind/*H*_E_ < 0.10 (invariable loci) or >1.0 (loci likely concerned by paralogy), which could bias the analyses, the expected *H*ind/*H*_E_ distribution was simulated under diploid, autotetraploid or autohexaploid inheritance and two inbreeding coefficients (0 and 0.5). Genotype calling was performed with the IteratePopStruct and GetWeightedMeanGenotypes functions. The IteratePopStruct function requires two parameters: (1) the overdispersion parameter, which was recalculated post-filtering, and (2) the inbreeding coefficient, which remains unknown. To determine a suitable value for the inbreeding coefficient, we first eliminate individuals exhibiting mean *H*ind/*H*_E_ values > 0.8 to avoid allopolyploid outliers with fixed heterozygosity which may incorrectly increase the index. We then simulated *H*ind/*H*_E_ across a range of inbreeding values. The observed distribution of *H*ind/*H*_E_ aligned most closely with the simulated distribution at an inbreeding of 0.2, and this value was selected as the optimal compromise for continuous genotype calling across all individuals.

### Genetic structure analyses

To characterize genetic structure accounting for uncertain ploidy levels, we employed a non-model-based clustering strategy. Euclidean genetic distances were calculated from continuous genotype data, followed by hierarchical clustering analysis (HCA) using Ward’s minimum variance algorithm. Cluster membership was defined by dendrogram truncation at levels maximizing congruence with prior taxonomic expectations. When groups with several taxa were obtained, each group was split into the corresponding taxa in relation and according to the clustering of the genotypes. The statistical robustness of the clustering analysis was tested with the permutational multivariate analysis of variance (permanova).

Group distinctiveness and potential admixture were first visualized with an heatmap based on a genotypic similarity matrice constructed as 1 – normalized Euclidean distances. To further evaluate groups separation, we performed Discriminant Analysis of Principal Components (DAPC) using the adegenet R package [51], with the groups previously defined as a priori discriminant factor. Cross-validation (xvalDapc) and optimization of the α-score (optim.a.score) were used to avoid overfitting. Where admixture signals were detected, Neighbor-net analyses [52] were performed on data subsets focusing only on the concerned genetics groups. Neighbor-net analyses were used to visualize relationships and conflicting genetic signals among closely related or potentially admixed groups.

In details, HCA was conducted with the ward algorithm, minimising within cluster variance on Euclidean distance between genotype (R dist and hclust functions). Cutree function was used to truncate and design clusters. Then the clusters were displayed on the dendrogram with the rec.hclust function. If a grouping according to taxa was observed in one cluster new groups were designed. The permanova were performed on the Euclidean genetic distance using the adonis2 function of vegan R package [53] and based on 1000 permutations. The heat map of genotypic similarity was computed on a matrix of genotypic similarities obtained after the following transformations: (i) division of Euclidean distances by their maximum distance to obtain values between 0 and 1 (normalized Euclidean distances) and (ii) the similarities were obtained by subtracting this distance from 1. A zero value was assigned to the diagonal of the matrix and then the heatmap was performed with the heat map function and the genotype ordered in row and columns according the dendrogram obtained above, allowing the observation of genetic groups on the heatmap. Colours were chosen to reflect genotypic similarities from grey (low values) to intense magenta (great similarities). With this method, isolated genetic groups, not connected among them by gene flow, will form square of genotypes aligned along the diagonal in a background of low similarities (grey), their colours will change according to the level of their divergence (magenta). However, in case of admixture between groups, patches or magenta, more or less intense (genetic similarity) will appear at the intersection of row and columns corresponding to the groups. The DAPC analysis was conducted with these groups as a priori factor (dapc function, adegenet R package) after having selected, with xvalDapc and the optim.a.score functions, the optimal number of principal components to avoid overfitting. For subset analyses, Euclidean genetic distances between genotypes were converted to nexus format with the write.nexus.dist function (phangorn R package; [54]) to draw network with the Neighbor-net method implemented in the SplitsTree App [52]. DAPC results were drawn with the ggplot2 R package [55].

### Plastid genome variant calling and phylogenetic inference

Given heterogeneous off-target plastid recovery among individuals, a variant-calling based phylogenetic approach was adopted. A reference was first assembled from one *R. florentii* specimen using GetOrganelle [56] with default parameters and annotated with GeSeq [57]. Reads were mapped to this plastome reference, and SNP calling was conducted with Freebayes in haploid mode (ploidy = 1). The resulting VCF was converted to FASTA format using vcf2phylip [58] and parsimony- informative sites were retained with ClipKit [59]. Sequences with > 30% missing data were discarded. Maximum likelihood phylogenetic trees were reconstructed in IQTREE [60] under a GTR + ASC substitution model, which is adapted to SNP data, with 100 parsimony starting trees and 1,000 unsuccessful iterations stop criterion. Node support was evaluated using ultrafast bootstrap (1,000 replicates; with -bnni option; [61]) and SH-aLRT likelihood ratio tests [62]. The link between nuclear and plastid metadata is given in S1 File.

## Results

### Nuclear SNP dataset assembly

Following quality filtering and the exclusion of low-coverage samples, 42 samples representative of the taxon diversity and geographical ranges were assembled and analysed with HybPiper. From 4 to 17 of million paired-end reads with an average of 12 were retained per sample after reads trimming. The on-target capture efficiency ranged from 16 to 76% with an average of 47%. Between 350 and 353 loci were recovered with an average of 9 loci (from one to 13) receiving a paralog warning based on sequence length criterion. Based on HybPiper recovery statistics, 331 loci were retained after discarding genes absent in >50% of samples (9 loci) and those with >2 paralog warnings (13 loci). After alignment, trimming, and manual curation, the concatenated supermatrix comprised approximately 316 kbp of nuclear sequence data. Consensus reference sequences derived from these loci were subsequently used as a custom reference for SNP discovery and genotype calling.

### Multilocus genotypes and heterozygocity analysis

After stringent filtering (MAF ≥ 0.01, *H*ind/*H*_E_ between 0.10 and 1.0), the final dataset contained 272 individuals genotyped at 8,168 high-confidence bi-allelic SNPs, with an overall missing data rate of 9%. Completeness heatmap (see Supporting information, S2 File) generated with vcfR revealed no systematic bias across individuals, confirming the dataset was suitable for downstream population-genetic analyses.

**Fig 2:**
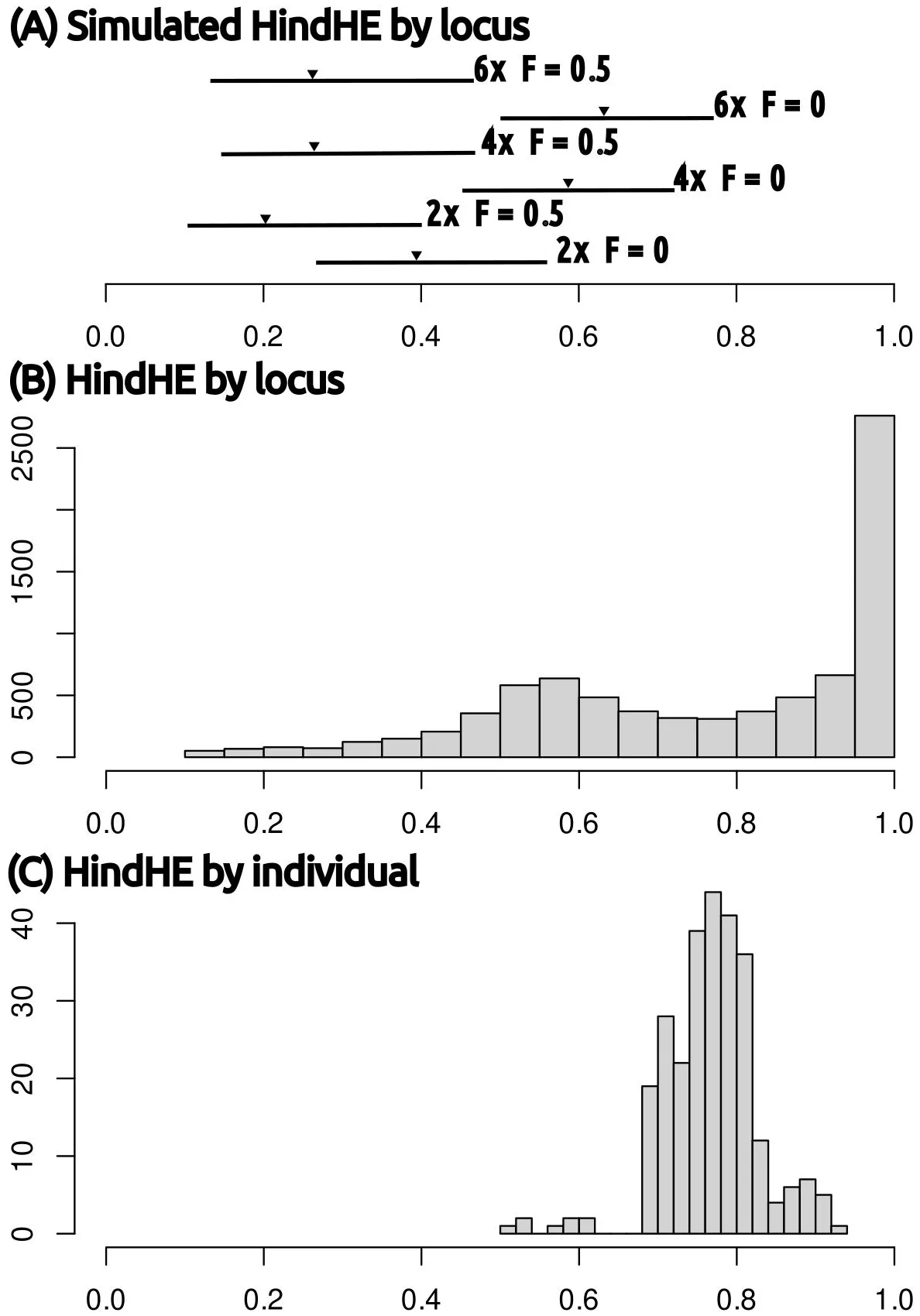
Heterozygosity assessment with the *H*ind/*H*_E_ analyses. (A) 95% CI and mean of values by locus after simulations according to diploid, autotetraploid or autohexaploid inheritance and two inbreeding values (F). (B) and (C) Histograms of observed values by locus or by individual.

Simulations of expected *H*ind/*H*_E_ under three modes of inheritance yielded a range between 0.10 and 0.77 (Fig 2A). The distribution of observed *H*ind/*H*_E_ across loci was bimodal, with a primary mode at approximately 0.56 and a secondary mode near 1.0 (Fig 2B). The observed peak at 0.56 is consistent with an autotetraploid inheritance model with a low inbreeding rate, whereas the peak near 1.0 is indicative of fixed heterozygosity, suggesting allopolyploid inheritance for a large subset of loci. At the individual level, *H*ind/*H*_E_ values ranged from 0.5 to 0.95 with a pronounced mode near 0.75 (Fig 2C), indicating that the majority of individuals exhibit inheritance patterns more compatible with allopolyploidy than with strict autopolyploidy.

### Genetic structure and clustering analyses

**Fig 3:**
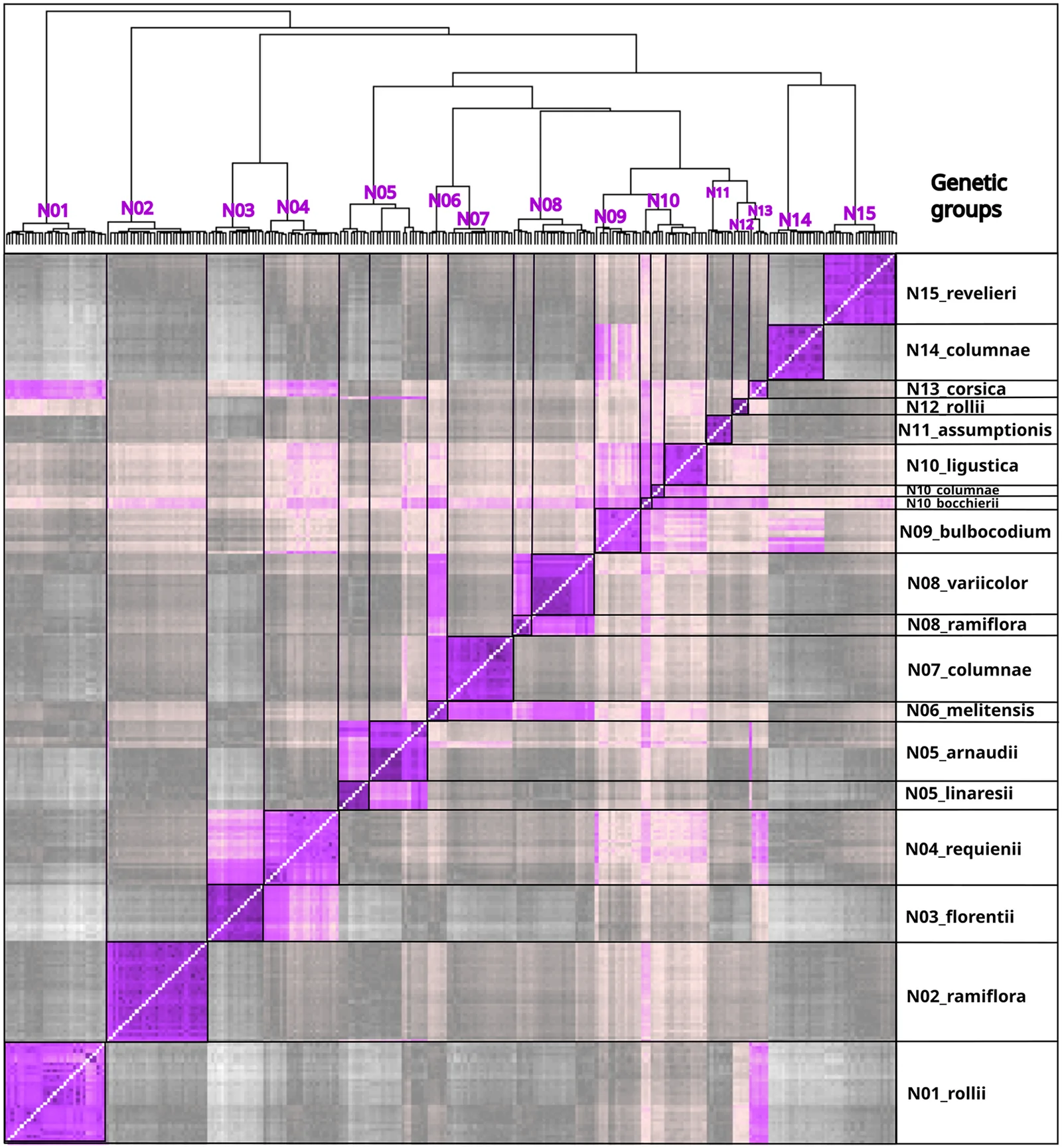
Genetic distinctiveness and admixture. Heatmap of similarities between 272 genotypes of 8168 biallelic SNPs for the *Romulea* taxa studied. Magenta intensity is proportional to genotypic similarities. Genotypes were organized according to a hierarchical clustering (Ward algorithm) of their dissimilarities, and 15 clusters were designed by truncation of the dendrogram, then some clusters were subdivided in respect to putative taxa, for finally designing 19 genetics groups. Permutations analysis revealed 37% of variance between groups.

Hierarchical clustering of Euclidean genetic distances delineated 15 primary clusters (Fig 3). Incorporation of taxonomic priors and examination of substructure led to the recognition of four additional subclusters, resulting in 19 genetically coherent groups. Permanova confirmed the overall significance of the clustering (p < 0.001, 999 permutations) with a R^2^ of 73% and 37% of the genetic variance assigned between groups.

Heatmap of genotypic similarity confirmed that most groups were strongly differentiated, forming distinct high-similarity blocks along the diagonal. Nevertheless, off-diagonal similarity signals were detected, indicative of gene flow or shared ancestry among several groups. Examples include pronounced similarity between N01 (*R. rollii*), N04 (*R. requienii*) and N13 (*R. corsica*), as well as between N06 (*R.* x *melitensis*) and its putative parental clusters N07 (*R. columnae*) and N08 (*R. variicolor*). Putative hybrids identified during fieldwork between *R. arnaudii* and *R. columnae* and between *R. arnaudii* and *R. rollii* were supported by their intermediate placement in the heatmap.

**Fig 4:**
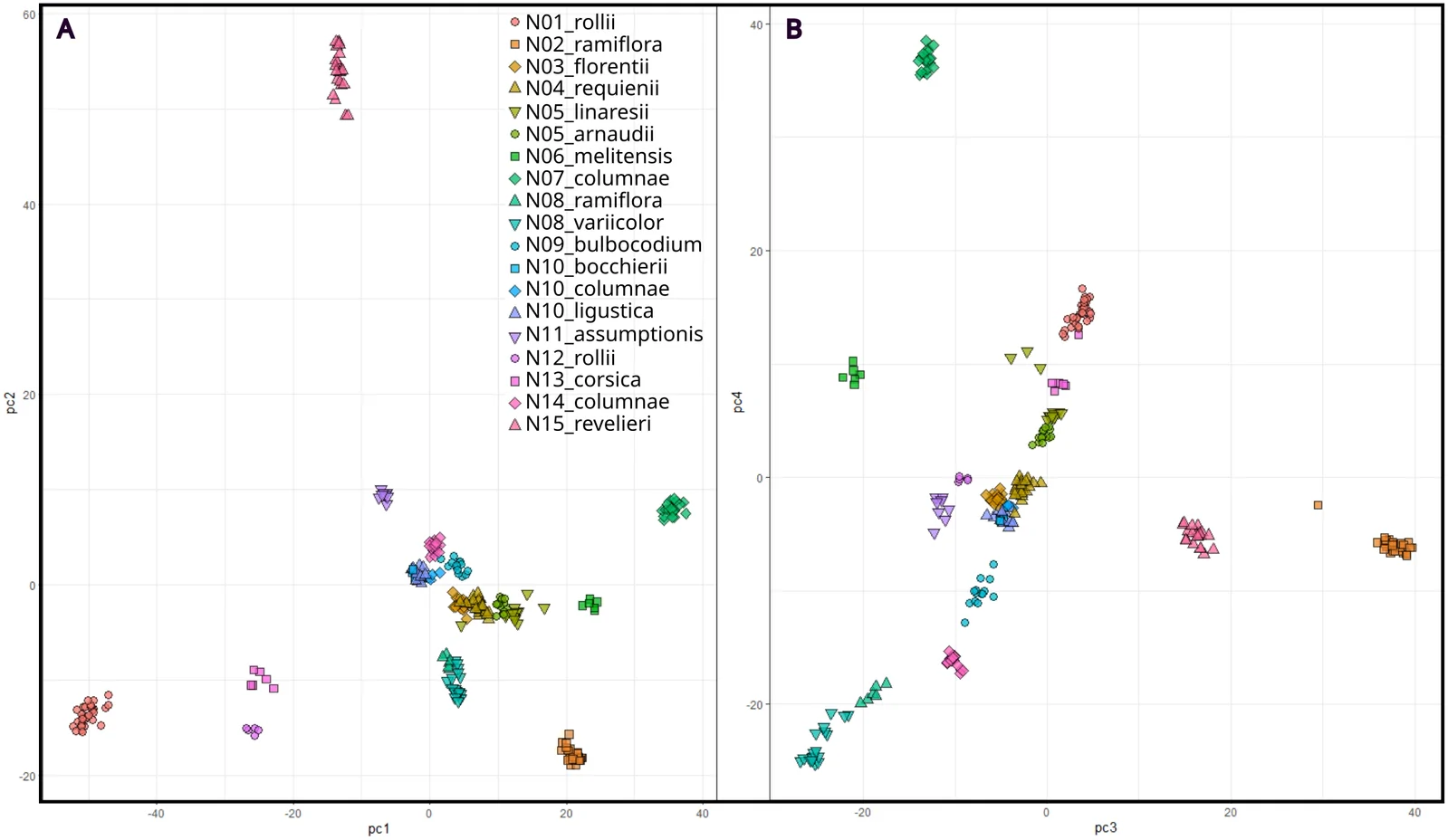
Genetic group separation visualization. Discriminant analysis of principal components (DAPC) of 272 genotypes of 8168 biallelic SNPs for the *Romulea* taxa studied. The genetic groups were used as *a priori* factors and the first 13 principal components (52% of the total variance) were chosen to conduct the DAPC. The first four DAPC axes are shown (A: axes 1 and 2; B: axes 3 and 4).

Following cross-validation to prevent overfitting, 13 principal components explaining 52% of the total genetic variance were retained for DAPC (Fig 4). The resulting discriminant functions separated the 19 genetic groups with variable degree of resolution. N01 (*R. rollii*), N02 (*R. ramiflora*), N07 (*R. columnae*), and N15 (*R. revelierei*) were among the most genetically isolated clusters. N08 (*R. variicolor* and *R. ramiflora*), N11 (*R. assumptionis*), and N14 (*R. columnae*) were also well-separated but closer, consistent with a more recent divergence or incomplete lineage sorting. The groups N03 (*R. florentii*), N04 (*R. requienii*), and N05 (*R. arnaudii* and *R. linaresii*) exhibited a partial overlap despite their geographical separation. Within N08 (*R. variicolor* and *R. ramiflora*) and within N10 (*R. ligustica*, *R. columnae* and *R. bocchierii*), genetic separation was minimal, consistent with either historical or ongoing admixture. N09 (*R. bulbocodium*) was isolated but always close and at intermediate distance between N10 and N14, suggesting a hybrid origin.

**Fig 5:**
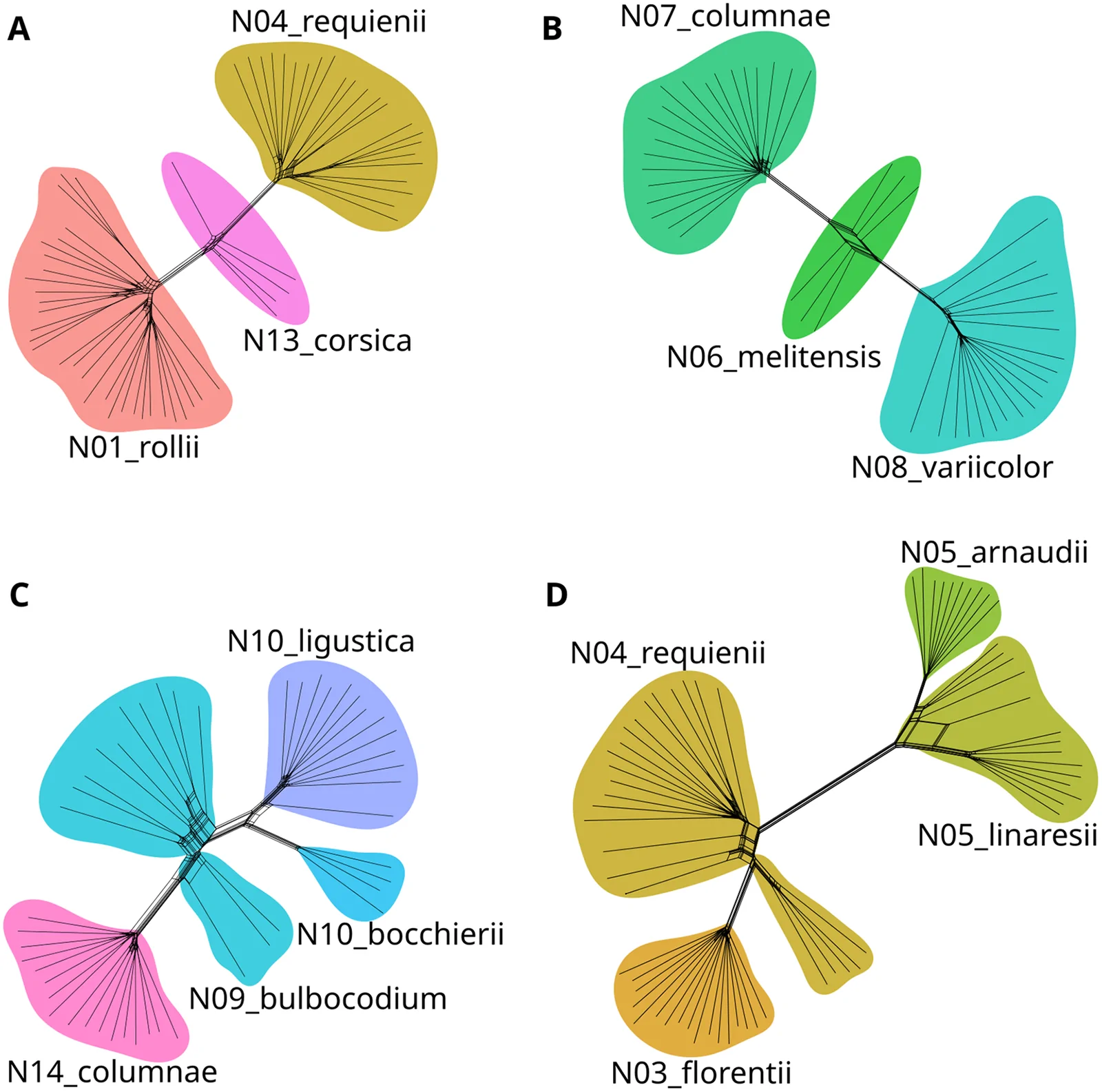
Genetic relationships between selected groups of *Romulea.* Neighbor-net networks, drawn at the same scale to allow comparison of branch lengths.

To further refine genetic groups and investigate admixture signatures, Neighbor-net analyses were conducted on selected groups (Fig 5). In the N01-N04-N13 triad, N13 (*R. corsica*) occupied an intermediate position between N01 (*R. rollii*) and N04 (*R. requienii*), suggesting its hybrid origin. Similarly, N06 (*R.* x *melitensis*) exhibited an intermediate position between N07 (*R. columnae*) and N08 (*R. variicolor*). N09 (*R. bulbocodium*) displayed an intermediate position between N10 (*R. ligustica*) and N14 (*R. columnae*) but some genotypes were closer to N10. The genotypes of *R. bocchierii* were in intermediate position between *R. ligustica* and *R. bulbocodium*, and one genotype of *R. ligustica* clustered with *R. bocchierii*. Although, N03 (*R. florentii*), N04 (*R. requienii*), and N05 (*R. arnaudii* and *R. linaresii*) display an important proximity according to DAPC (Fig 4), Neighbor-net analyses (Fig 5) support that they are forming two isolated genetic groups. Despite their current geographical isolation, the Nnet network suggests that *R. arnaudii* and *R. florentii* derive from *R. linaresii* and *R. requienii*, respectively.

### Plastid phylogenomics

The plastome assembled for *R. florentii,* having a length of 151 kbp, was used for variant calling (Supporting information, S2 File). Variant-based plastid phylogeny (see the tree in Supporting information, S3 File) was reconstructed from 262 haplotypes across 965 parsimony-informative sites, with an overall missing data rate of 4%. The resulting Mediterranean clade was strongly supported (100% node support for ultrafast bootstrap and SH-aLRT likelihood ratio) as monophyletic and contained 798 parsimony informative sites after excluding South African haplotypes. Maximum likelihood analyses resolved 23 well-supported Mediterranean clades (support > 90% for ultrafast bootstrap and/or SH-aLRT likelihood ratio). These 23 plastid clades were numerically indexed for cross-comparison with nuclear genetic groups. The haplotypes were partitioned into six major lineages, one of which encompassed clades 6–23, though the relationships among these internal clades remained poorly resolved. Although terminal clades exhibited robust node support, several deeper nodes lacked robustness, resulting in a star-like diversification pattern consistent with rapid radiation.

### Review of *Romulea* genetic groups

The nineteen genetic groups, based on nuclear genotypic distances, were matched to plastid haplotype clades, taxonomic assignments, and geographic areas (Table 2). We review these groups individually, focusing on their distinctiveness and correspondence with morphological identifications.

**Table 2:** Review of plastid data, taxonomy and distribution for 19 nuclear genetic groups of *Romulea.*

Genetic_groups	n	Plastid_clades *	Taxa	Capr.	Cors.	Hyer.	Ligu.	Malt.	Mino.	Prov.	Sard.	Sici.
**N01_rollii**	31	**P14 (28**), P23	*R. rollii* (31)		6	6			3	10	3	3
**N02_ramiflora**	31	P03	*R. ramiflora* (29), *R. columnae* x *ramiflora*, *R.* aff *ramiflora*		9	1			4	11	3	3
**N03_florentii**	17	**P08**	*R. florentii* (17)			12				5		
**N04_requienii**	23	P11 **(10)**, P10, P09, P08	*R. requienii* (23)		14						9	
**N05_arnaudii**	13	**P21 (10)**, P19	*R. arnaudii* (10), *R. arnaudii* x *columnae*							13		
**N05_linaresii**	14	P20 **(12)**, P21,	*R. linaresii* (14)									14
**N06_melitensis**	6	P18	*R.* x *melitensis* (6)					6				
**N07_columnae**	20	**P18 (14)**, P19, P05	*R. columnae* (16), *R. columnae* x *ramiflora*, *R.* aff *ramiflora*		2		1	2	7	7	1	
**N08_ramiflora**	6	**P23**	*R. ramiflora*				3			2		1
**N08_variicolor**	19	**P23 (14)**, P22, P18, P04	*R. variicolor* (18), *R. columnae*					6				13
**N09_bulbocodium**	14	P13 **(4),** P15, P12, P04	*R. bulbocodium* (13), *R. corsica*		1		4				3	6
**N10_bocchierii**	3	**P01**	*R. bocchierii* (3)								3	
**N10_columnae**	3	P05, P19	*R. columnae* (2), *R. columnae* x *ramiflora*				2			1		
**N10_ligustica**	14	P07 **(13)**, P01	*R. ligustica* (14)		3		4				7	
**N11_assumptionis**	8	P06	*R. assumptionis* (8)			3			5			
**N12_rollii**	5	**P14**	*R. rollii* (5)			5						
**N13_corsica**	6	**P14 (4),** P02	*R. corsica* (5), *R. arnaudii_*x*_rollii*		5					1		
**N14_columnae**	17	**P15 (14)**, P18	*R. columnae* (17)		9						8	
**N15_revelieri**	22	P17 **(13)**, P16, P15, P14, P20	*R. revelieri* (18), *R. insularis, R.* x *jordanii*	2	17						3	

* in bold the main plastid clade with its number of samples, in underlined when they are private.

**N01.** This group is distributed across six areas and corresponds exclusively to *R. rollii*. It is associated predominantly with plastid clade P14. P14 is also present in *R. corsica,* suggesting *R. rollii* as the maternal parent of this taxon.

**N02.** Found in six areas, this group includes *R. ramiflora*, *R. aff ramiflora* from Minorca, and hybrids involving *R. columnae*. It is characterized by exclusive association with plastid clade P3. Most individuals identified as *R. ramiflora* fall within this group.

**N03 and N04.** These two groups are closely related. N03 corresponds to *R. florentii,* endemic to Provence and the Hyères Islands, while N04 corresponds exclusively to *R. requienii*, endemic to Corsica and Sardinia. Nnet analysis shows that *R. florentii* derives from *R. requienii*. Both share plastid lineages, although N04 exhibits greater nuclear genetic diversity. *Romulea requienii* functions as the paternal parent of *R. corsica*.

**N05.** This group encompasses *R. arnaudii* and *R. linaresii*, narrow endemics of Provence and northern Sicily, respectively. Their differentiation remains incomplete in all analyses (Fig 4 and Fig 5). Two closely related plastid clades are specific to N05: P20, which is unique to *R. linaresii*, and P21, which is shared between *R. linaresii* and *R. arnaudii*. Within P21, the haplotypes of *R. arnaudii* form a monophyletic group, supported by bootstrap and SH-aLRT values of 88/88.

**N06.** This group corresponds to *R.* x *melitensis*, a narrow endemic hybrid of Malta. Nuclear clustering places it in intermediate position between N07 (*R. columnae*) and N08 (*R.* *variicolor*), consistent with the hypothesis of hybrid origin involving these two parental lineages.

**N07.** Genotypes within this group were identified primarily as *R. columnae*, with a minority identified as *R.* aff. *ramiflora* in Minorca. N07 is found in six areas, with main sampling in Provence and Minorca. It contains three plastid clades, among which P18 is the most common and specific to N07 and N06 suggesting N07 as the maternal lineage of *R.* x *melitensis*.

**N08.** This group includes all *R. variicolor* genotypes from Sicily and Malta, a subset of *R. ramiflora* individuals from multiple localities, and some *R. columnae* individuals. Within N08, all *R. ramiflora* and a majority of *R. variicolor* are associated with plastid clade P23, whereas clade P22 is restricted to *R. variicolor*. *R. ramiflora* genotypes in this group are genetically closer to *R. variicolor* than to the *R. ramiflora* individuals of N02.

**N09.** This group corresponds to *R. bulbocodium* and includes a single genotype of *R. corsica*. Heatmap and DAPC (Figs 3, 4) indicate that *R. bulbocodium* shares affinities with both N10 (*R. ligustica*) and N14 (*R. columnae*), suggesting these lineages as potential parental taxa. Plastid diversity is high in N09, with fourteen sequenced samples revealing a polyphyletic pattern.

**N10.** This group contains *R. ligustica* genotypes, which form a distinct group and possess a private plastid lineage. *Romulea ligustica* occurs in three regions: Liguria, Corsica and Sardinia. Additionally, three genotypes clustering near *R. ligustica* were morphologically identified as *R. columnae* (two individuals from Liguria) or as a hybrid between *R. ramiflora* and *R. columnae* (one individual from Provence). *R. bocchierii,* endemic of Sardinia, is close to *R. ligustica* and *R. bulbocodium*, but it has its own plastid lineage (P01) which is also the most basal in plastid phylogeny.

**N11.** This group includes *R. assumptionis* genotypes collected in Minorca and the Hyères islands. They are also identified by the plastid clade P06. Plastid relationship is consistent with derivation of French haplotypes from Minorcan populations.

**N12.** Although these genotypes of Hyères islands, *identified as R. rollii*, share strong nuclear similarity with N01 (*R. rollii*) and belong to the same plastid lineage, they consistently form a separate genetic group in all analyses.

**N13.** This group is composed mainly of *R. corsica*. Analyses indicate a hybrid origin with *R. requienii* and *R. rollii* as parental taxa. Four, out of six plastid haplotypes, belong to the *R. rollii* lineage, while two form a distinct lineage near the base of the plastid phylogenetic tree. N13 also contains a genotype which is probably and hybrid between *R. arnaudii* and *R. rollii*.

**N14.** This group corresponds to a second genetic group of *R. columnae*. Genotypes are clearly discriminated but remain genetically close to N09 (*R. bulbocodium*) and N10 (*R. ligustica*), with which hybridization has occurred. N14 includes populations from Corsica and Sardinia, and most individuals carry plastid clade P15, which is also shared with *R. bulbocodium* (N09) and *R. revelieri* (N15).

**N15.** This group corresponds primarily to *R. revelieri*, endemic of Corsica and Sardinia, as well as *R.* x *jordanii* (a putative hybrid between *R. revelieri* and *R. ramiflora*) and *R. insularis* (endemic to Capraia island). The latter two fall entirely within the genetic diversity of *R. revelieri*. N15 is associated with four plastid clades, two of which are exclusive to *R. revelieri* and two that are shared with other taxa.

## Discussion

Species constitute the fundamental operational units of biodiversity research, and their accurate delimitation is essential for linking observed diversity patterns to underlying evolutionary processes [63–65]. Our multilocus nuclear and plastid datasets together provide the first comprehensive molecular framework for *Romulea* of the Mediterranean region, enabling an evaluation of species boundaries and hybridization in this genus. While our results reveal a generally strong concordance between genetic groups and current taxonomic concepts, they also highlight instances of hybridization including plastid capture, indication that species boundaries in *Romulea* are permeable.

### Evidences for hybridization

The distribution of *H*ind/*H*_E_ across loci and individuals was incompatible with strictly diploid or autopolyploid inheritance models. The high number of loci having *H*ind/*H*_E_ value near one is indicative of a fixed heterozygosity which is consistent with the hypothesis of allopolyploidy events in the evolutionary history of the Mediterranean *Romulea*. Moreover, several taxa (e.g., *R.* x *melitensis*, *R. corsica, R. bocchierii* and *R. bulbocodium*) exhibit genetic clustering consistent with hybrid origins, further supporting the role of hybridization in evolution of Mediterranean *Romulea*. In *R.* x *melitensis*, the nuclear data and plastid identity converge on a biparental origin involving N07 (*R. columnae*) and N08 (*R. variicolor*). By contrast, *R. corsica* and especially *R. bulbocodium* appear to have more complex hybrid origins, possibly involving multiple parental lineages and repeated hybridization events, as suggested by their association with several plastid clades. The Neighbor-net analysis is also consistent with the hybrid origin of the narrow Sardinian endemic *R. bocchierii* [20], involving *R. bulbocodium* and *R. ligustica,* however it has its own plastid haplotype and is also closely related to some *R. columnae* genotypes, suggesting a more complicated origin, possibly involving more than two progenitors. Multivariate analyses are suggesting that *R. columnae* was involved in several cases of hybridization. Interestingly, *R. columnae* has a very different morphology (small white flowers) from most other species of *Romulea*.

Our assessment of the role of hybridization in the evolution of Mediterranean *Romulea* is exacerbated by at least five cases of plastid capture [66], where the plastid haplotype of a taxon did not match its nuclear genetic background. Examples include Sicilian populations of *R. rollii* sharing the P23 plastid clade with *R. variicolor*, few individuals of *R. revelieri* having plastid haplotypes shared with other taxa (P14, P15, and P19), or certain *R. arnaudii* individuals carrying plastid haplotypes characteristic of *R. columnae* (P19). It is remarkable that these plastid captures were observed between populations belonging to well differentiated genetic groups. These findings suggest that introgression has been active during the evolutionary history of Mediterranean *Romulea* taxa.

### Species boundaries and taxonomic implications

Our results confirm the distinctiveness of four taxa (*R. assumptionis* (N11), *R. revelieri* (N15), *R. ligustica* (N10), and *R. rollii* (N01)) which are supported as independently evolving lineages by both nuclear and plastid data. These taxa can thus be considered robust species hypotheses under a genotypic cluster concept of species delimitation [67]. This concept applying population genetics to species delimitation was proven successful with appropriate sampling [29,68]. *Romulea revelieri*, *R. ligustica* and *R. rollii* are tetraploid whereas *R. assumptionis* is an hexaploid [20]. N02 (*R. ramiflora*) and N07 (*R. columnae*) are clearly isolated genetic groups, which represent a substantial part of the genetic variance analysed here, and they are also forming robust species hypothesis. However, other groups were identified as *R. ramiflora* or *R. columnae*. *Romulea ramiflora* forms two nuclear groups, only one of which (N02) exhibits clear plastid and nuclear distinctiveness. The second group (N08) was rarely observed in 3 areas (Provence, Liguria and Sicily). It is closely related to *R. variicolor*, abundant in Malta and very rare in Sicily, with genetic overlap and shared plastid identity. More data are necessary to decipher its role in the origin of *R. variicolor*. *Romulea columnae* is represented by three separate genetic groups (N07, N10, N14), suggesting that what is currently treated as a single species may instead constitute a complex of partially isolated lineages, which frequently hybridise with other genetic groups. Given its wide distribution across the Mediterranean, the study of *R. columnae* should be expanded to include additional populations. The correlation of these different lineages with the karyological variability [20] merits further investigation.

In addition, our study questions the status of several narrow endemics. *Romulea* x *jordanii* (endemic of Corsica) and *R. insularis* (endemic of Capraia Island, Tuscan Archipelago) are genetically indistinguishable from *R. revelieri* (common in Corsica but rare in Sardinia), supporting the hypothesis that they may represent intraspecific polymorphism or karyotype variation (*R. insularis* being pentaploid [20]) rather than distinct species. *Romulea* x *jordanii* is usually considered as an hybrid between *R. revelierei* and *R. ramiflora*. It appears in mixed populations. It is not always easy to distinguish, and the only specimen we were able to find was not typical (pale yellow corolla throat). It is possible that it was a somewhat unusual *R. revelierei*. Although close in our analyses, the two pairs of taxa (*R. arnaudii* / *R. linaresii*; *R. florentii* / *R. requienii*) form two independent genetic groups that are well distinct from the other genetic groups. They should be considered as two robust species hypotheses but, currently, both contain two taxa. *Romulea arnaudii* (endemic from Saint-Tropez, Provence) and *R. linaresii sensu stricto* (endemic of N Sicily) despite being separated by a large geographic distance are still very close since they share a single genetic group (N05) and plastid haplotypes. Their plastid differentiation is limited to private haplotypes, suggesting that they recently diverged, possibly during the late Pleistocene. The differentiation between *R. requienii* (Corso-Sardinian endemic) and *R. florentii* (endemic from Hyères Islands and Cap Bénat, Provence) is more evident. However, the latter is part of the genetic diversity of *R. requienii*, suggesting incomplete lineage sorting. For both cases, investigating the causes of their geographical isolation with the support of divergence time analyses, will be necessary to definitively assess their taxonomic status. *Romulea bocchierii*, a very rare endemic to Sardinia with a pentaploid karyotype, has a unique plastid lineage that branches at the root of the plastid phylogenetic tree and shows close relationships with three genetic groups. This underlines its distinctiveness and originality. The sole population of *R. bocchierii* grows on a plateau on substrate originating from Paleozoic metamorphic rocks. Currently, the only other species found in the same habitat is *R. ligustica*. The currently known populations of *R. bulbocodium* in Sardinia are more than 100 km away from the population of *R. bocchierii*. *Romulea variicolor* is also supported by molecular data, but its close relationship with one group of *R. ramiflora* need further analyses to decipher the distinctiveness of this taxon. Finally, two rare and endemic taxa, *R. corsica* and *R.* x *melitensis*, are also supported as well as their hybrid origin.

In summary, our study shows that genetic boundaries are robust enough to propose species hypotheses despite evidences of hybridization, which undoubtedly had a significant contribution to the diversification of the *Romulea* genus in the Mediterranean. After this first assessment, it remains an important issue concerning *R. bulbocodium*. This aggregate exhibits great variability, but it is still poorly understood in terms of taxonomy. Despite its limited sampling in this study, it is one of the most common taxa in the Mediterranean. Our first results suggest that *R. bulbocodium* establishes a genetic link with several genetic groups and its intermediate position between *R. ligustica* (N10) and *R. columnae* (N14), revealed by genotypic similarities, is clearly challenged by its wide plastid diversity. Further studies encompassing more western Mediterranean and Atlantic populations are needed to better understand the evolutionary patterns and the links between the diverse taxonomic entities described within *R. bulbocodium* aggregate.

### Biogeographic and evolutionary insights

Because of their occurrence throughout the Mediterranean basin and on all the major islands, *Romulea* taxa constitute a fascinating model for documenting the complex historical biogeography of this area. In this first molecular study, the genetic data reveal striking patterns of evolutionary links among populations separated by large geographic distances and insular barriers. This pattern of relatedness despite isolation is a recognised feature of the Tyrrhenian biogeography, with numerous endemic taxa shared between the various islands for plants [28] and animals [69]. To interpret these similarities in terms of connectivity or common ancestry predating the separation of the islands from the mainland, it is necessary to compare the likely times of divergence of the populations with the geological history of their areas of occurrence. Certain studies have revealed divergence times compatible with paleogeographic phenomena linked to the separation of the Proto-Ligurian massif from the continent and the rotation and isolation of Corsica and Sardinia [70]. However, other studies have revealed more recent divergences, suggesting long-distance dispersal [71–73] or the maintenance of a physical connection, still hypothetical, between the islands and the continent until the end of the Miocene [74]. Some pairs of lineages, which are still very close, such as *R. florentii* versus *R. requienii*, or *R. arnaudii* versus *R. linaresii*, or even the two current areas of presence (Balearic Islands and Hyères Islands in Provence) for *R. assumptionis*, clearly raise doubts about isolation events more ancient than the Pliocene. The observed star-like topology of the plastid phylogeny further supports a scenario of Pleistocene diversification. Thus, the present study opens an important issue for performing divergence time analyses of the several genetic groups revealed here in relation to the palaeogeography of the Mediterranean Basin.

### Challenges in the molecular assessment of the overlooked genus *Romulea*

Several challenges were met in this molecular assessment of the Mediterranean genus *Romulea*. Gaps in the Linnaean classification were partially addressed through collaborative efforts among botanists based on field photographs, an approach unfeasible with herbarium specimens alone. A second, unforeseen challenge emerged during a pilot study: phylogenetic analyses of nuclear data assembled with HybPiper revealed weak node robustness. Without prior knowledge of ploidy levels or the origins of potential allopolyploids, applying methods to separate gene copies was difficult [75]. Our findings confirm this limitation, as they indicate that hybridization is concerning almost all Mediterranean *Romulea* species. To address these challenges, one promising direction is to identify diploid ancestors and generate long-read data. This would enable haplotype phasing during assembly and the isolation of homeologs. The absence of a reference genome for variant identification remains a limitation, however in a genus where hybridization and allopolyploidy is so prevalent, the utility of a single reference genome is uncertain. Consequently, our strategy, consisting of using reduced-cost genomic sequencing, constructing a custom reference, performing continuous genotyping and confronting taxa to genotypes clusters, may represent a pragmatic compromise; it provided sufficient genetic information for an initial assessment of Mediterranean *Romulea* while balancing feasibility and cost.

## Conclusions and future directions

Our study provides the first genome-scale assessment of *Romulea* taxa in the Mediterranean Basin, uncovering a complex interplay of polyploidy, hybridization, plastid capture, and incomplete lineage sorting. Our findings underscore the need for more extensive genomic studies. These investigations should initially verify the allopolyploid nature of Mediterranean *Romulea*, followed by the identification of the homeologs genomes and their ancestry. This would allow to refine properly the relationships between the different taxa. The aim of molecular identification of taxa has offered opportunities for exchange and discussion between partners from several countries on the validity of taxa in Mediterranean *Romulea*. It appears that several genetic groups, and several taxa, are constituting robust species hypotheses, opening perspectives for *Romulea* systematics and conservation in the Mediterranean.

## Supporting information

S1 File Full metadata of all genotypes. The genetic data and scripts files to perform analyses are available on Zenodo (https://zenodo.org/records/21872209).
(CSV)

S2 File Supplementary molecular analyses. Data completeness of 272 *Romulea* genotypes. Plastome map of Romulea florentii.
(DOCX)

S3 File Maximum likelihood tree of 262 plastid haplotypes. IQtree ML tree based on 965 parsimony informative sites from the whole plastome.
(PDF)
