# Supplementary material for: Molecular identification and diversity assessment of Tyrrhenian Romulea species (Iridaceae)
Source: PLoS One. 2026 Sep 15;21(9):e0358245. doi: 10.1371/journal.pone.0358245 (PMC13577335; doi:10.1371/journal.pone.0358245)
Supplement: Supplementary file 1 [file pone.0358245.s003.pdf]

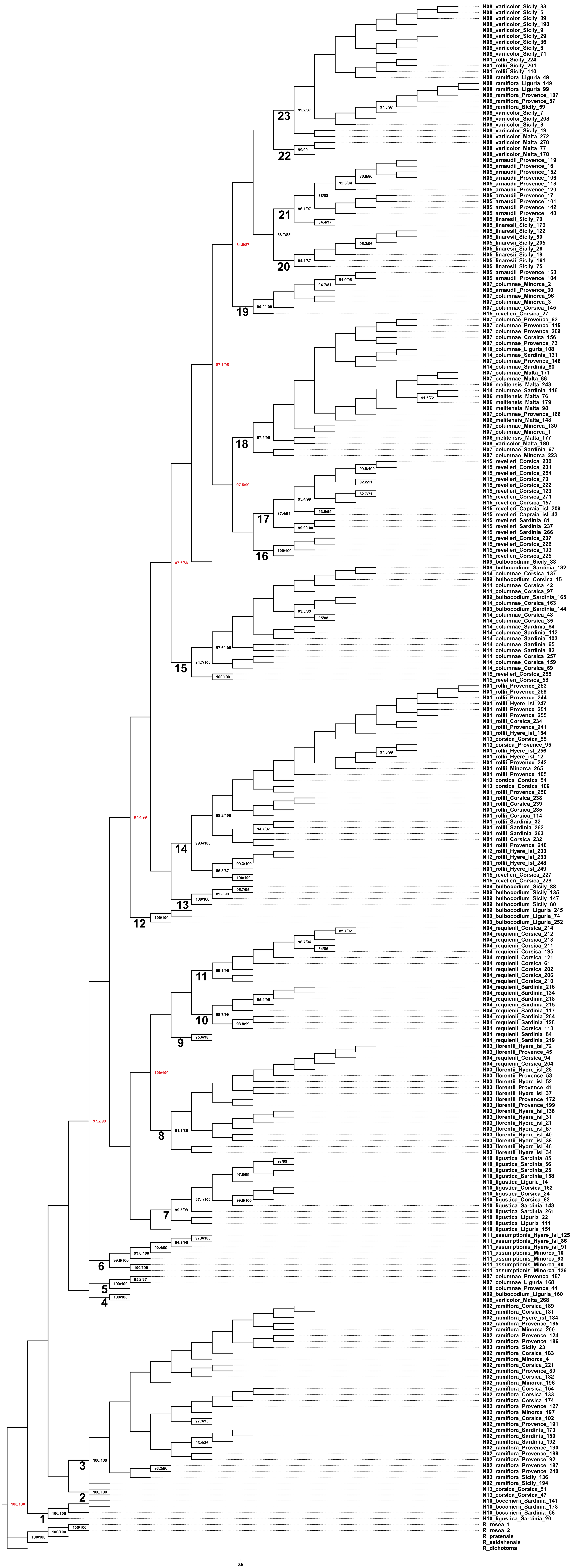
N08_variicolor_Sicily_33
N08_variicolor_Sicily_5
N08_variicolor_Sicily_39
N08_variicolor_Sicily_198
N08_variicolor_Sicily_9
N08_variicolor_Sicily_29
N08_variicolor_Sicily_36
N08_variicolor_Sicily_6
N08_variicolor_Sicily_71
N01_rollii_Sicily_224
N01_rollii_Sicily_201
N01_rollii_Sicily_110
N08_ramiflora_Liguria_49
N08_ramiflora_Liguria_149
N08_ramiflora_Liguria_99
N08_ramiflora_Provence_107
N08_ramiflora_Provence_57
N08_ramiflora_Sicily_59
N08_variicolor_Sicily_7
N08_variicolor_Sicily_208
N08_variicolor_Sicily_8
N08_variicolor_Sicily_19
N08_variicolor_Malta_272
N08_variicolor_Malta_270
N08_variicolor_Malta_77
N08_variicolor_Malta_170
N05_arnaudii_Provence_119
N05_arnaudii_Provence_16
N05_arnaudii_Provence_152
N05_arnaudii_Provence_106
N05_arnaudii_Provence_118
N05_arnaudii_Provence_120
N05_arnaudii_Provence_17
N05_arnaudii_Provence_101
N05_arnaudii_Provence_142
N05_arnaudii_Provence_140
N05_linaresii_Sicily_70
N05_linaresii_Sicily_176
N05_linaresii_Sicily_122
N05_linaresii_Sicily_50
N05_linaresii_Sicily_205
N05_linaresii_Sicily_26
N05_linaresii_Sicily_18
N05_linaresii_Sicily_161
N05_linaresii_Sicily_75
N05_arnaudii_Provence_153
N05_arnaudii_Provence_104
N07_columnae_Minorca_2
N05_arnaudii_Provence_30
N07_columnae_Minorca_96
N07_columnae_Minorca_3
N07_columnae_Corsica_145
N15_revelieri_Corsica_27
N07_columnae_Provence_62
N07_columnae_Provence_115
N07_columnae_Provence_269
N07_columnae_Corsica_156
N07_columnae_Provence_73
N10_columnae_Liguria_108
N14_columnae_Sardinia_131
N07_columnae_Provence_146
N14_columnae_Sardinia_60
N07_columnae_Malta_171
N07_columnae_Malta_66
N06_melitensis_Malta_243
N14_columnae_Sardinia_116
N06_melitensis_Malta_76
N06_melitensis_Malta_179
N06_melitensis_Malta_98
N07_columnae_Provence_166
N06_melitensis_Malta_148
N07_columnae_Minorca_130
N07_columnae_Minorca_1
N06_melitensis_Malta_177
N08_variicolor_Malta_180
N07_columnae_Sardinia_67
N07_columnae_Minorca_223
N15_revelieri_Corsica_230
N15_revelieri_Corsica_231
N15_revelieri_Corsica_254
N15_revelieri_Corsica_79
N15_revelieri_Corsica_222
N15_revelieri_Corsica_129
N15_revelieri_Corsica_271
N15_revelieri_Corsica_157
N15_revelieri_Capraia_isl_209
N15_revelieri_Capraia_isl_43
N15_revelieri_Sardinia_81
N15_revelieri_Sardinia_237
N15_revelieri_Sardinia_266
N15_revelieri_Corsica_207
N15_revelieri_Corsica_226
N15_revelieri_Corsica_193
N15_revelieri_Corsica_225
N09_bulbocodium_Sicily_83
N09_bulbocodium_Sardinia_132
N14_columnae_Corsica_137
N09_bulbocodium_Corsica_15
N14_columnae_Corsica_42
N14_columnae_Corsica_97
N09_bulbocodium_Sardinia_165
N14_columnae_Corsica_163
N09_bulbocodium_Sardinia_144
N14_columnae_Corsica_48
N14_columnae_Corsica_35
N14_columnae_Sardinia_64
N14_columnae_Sardinia_112
N14_columnae_Sardinia_103
N14_columnae_Sardinia_65
N14_columnae_Sardinia_82
N14_columnae_Corsica_257
N14_columnae_Corsica_159
N14_columnae_Corsica_69
N15_revelieri_Corsica_258
N15_revelieri_Corsica_58
N01_rollii_Provence_253
N01_rollii_Provence_259
N01_rollii_Provence_244
N01_rollii_Hyere_isl_247
N01_rollii_Provence_251
N01_rollii_Provence_255
N01_rollii_Corsica_234
N01_rollii_Provence_241
N01_rollii_Hyere_isl_164
N13_corsica_Corsica_55
N13_corsica_Provence_95
N01_rollii_Hyere_isl_256
N01_rollii_Hyere_isl_12
N01_rollii_Provence_242
N01_rollii_Minorca_265
N01_rollii_Provence_105
N13_corsica_Corsica_54
N13_corsica_Corsica_109
N01_rollii_Provence_250
N01_rollii_Corsica_238
N01_rollii_Corsica_239
N01_rollii_Corsica_235
N01_rollii_Corsica_114
N01_rollii_Sardinia_32
N01_rollii_Sardinia_262
N01_rollii_Sardinia_263
N01_rollii_Corsica_232
N01_rollii_Provence_246
N12_rollii_Hyere_isl_203
N12_rollii_Hyere_isl_233
N01_rollii_Hyere_isl_248
N01_rollii_Hyere_isl_249
N15_revelieri_Corsica_227
N15_revelieri_Corsica_228
N09_bulbocodium_Sicily_88
N09_bulbocodium_Sicily_135
N09_bulbocodium_Sicily_147
N09_bulbocodium_Sicily_80
N09_bulbocodium_Liguria_245
N09_bulbocodium_Liguria_74
N09_bulbocodium_Liguria_252
N04_requienii_Corsica_214
N04_requienii_Corsica_212
N04_requienii_Corsica_213
N04_requienii_Corsica_211
N04_requienii_Corsica_195
N04_requienii_Corsica_121
N04_requienii_Corsica_61
N04_requienii_Corsica_202
N04_requienii_Corsica_206
N04_requienii_Corsica_210
N04_requienii_Sardinia_216
N04_requienii_Sardinia_134
N04_requienii_Sardinia_218
N04_requienii_Sardinia_215
N04_requienii_Sardinia_117
N04_requienii_Sardinia_264
N04_requienii_Sardinia_128
N04_requienii_Corsica_113
N04_requienii_Sardinia_84
N04_requienii_Sardinia_219
N03_florentii_Hyere_isl_72
N03_florentii_Provence_45
N04_requienii_Corsica_94
N04_requienii_Corsica_204
N03_florentii_Hyere_isl_28
N03_florentii_Provence_53
N03_florentii_Hyere_isl_52
N03_florentii_Provence_41
N03_florentii_Hyere_isl_37
N03_florentii_Provence_172
N03_florentii_Provence_199
N03_florentii_Hyere_isl_138
N03_florentii_Hyere_isl_31
N03_florentii_Hyere_isl_21
N03_florentii_Hyere_isl_87
N03_florentii_Hyere_isl_40
N03_florentii_Hyere_isl_38
N03_florentii_Hyere_isl_46
N03_florentii_Hyere_isl_34
N10_ligustica_Sardinia_85
N10_ligustica_Sardinia_56
N10_ligustica_Sardinia_25
N10_ligustica_Sardinia_158
N10_ligustica_Liguria_14
N10_ligustica_Corsica_162
N10_ligustica_Corsica_24
N10_ligustica_Corsica_63
N10_ligustica_Sardinia_143
N10_ligustica_Sardinia_261
N10_ligustica_Liguria_22
N10_ligustica_Liguria_111
N10_ligustica_Liguria_151
N11_assumptionis_Hyere_isl_125
N11_assumptionis_Hyere_isl_86
N11_assumptionis_Hyere_isl_91
N11_assumptionis_Minorca_10
N11_assumptionis_Minorca_93
N11_assumptionis_Minorca_90
N11_assumptionis_Minorca_126
N07_columnae_Provence_167
N07_columnae_Liguria_168
N10_columnae_Provence_44
N09_bulbocodium_Liguria_160
N08_variicolor_Malta_268
N02_ramiflora_Corsica_189
N02_ramiflora_Corsica_181
N02_ramiflora_Hyere_isl_184
N02_ramiflora_Provence_185
N02_ramiflora_Minorca_200
N02_ramiflora_Provence_124
N02_ramiflora_Provence_186
N02_ramiflora_Sicily_23
N02_ramiflora_Corsica_183
N02_ramiflora_Minorca_4
N02_ramiflora_Corsica_221
N02_ramiflora_Provence_89
N02_ramiflora_Corsica_182
N02_ramiflora_Minorca_196
N02_ramiflora_Corsica_154
N02_ramiflora_Corsica_133
N02_ramiflora_Corsica_174
N02_ramiflora_Provence_127
N02_ramiflora_Minorca_197
N02_ramiflora_Corsica_102
N02_ramiflora_Provence_191
N02_ramiflora_Sardinia_173
N02_ramiflora_Sardinia_150
N02_ramiflora_Sardinia_192
N02_ramiflora_Provence_190
N02_ramiflora_Provence_188
N02_ramiflora_Provence_92
N02_ramiflora_Provence_187
N02_ramiflora_Provence_240
N02_ramiflora_Sicily_136
N02_ramiflora_Sicily_194
N13_corsica_Corsica_51
N13_corsica_Corsica_47
N10_bocchierii_Sardinia_141
N10_bocchierii_Sardinia_178
N10_bocchierii_Sardinia_68
N10_ligustica_Sardinia_20
R_rosea_1
R_rosea_2
R_pratensis
R_saldahensis
R_dichotoma
