# Supplementary material for: Molecular identification and diversity assessment of Tyrrhenian Romulea species (Iridaceae)
Source: PLoS One. 2026 Sep 15;21(9):e0358245. doi: 10.1371/journal.pone.0358245 (PMC13577335; doi:10.1371/journal.pone.0358245)
Supplement: Supplementary file 2 [file pone.0358245.s002.docx]

**Supplementary material**

**Title: Molecular identification and diversity assessment of Tyrrhenian *Romulea* species**

**Fig. S1** Data completeness of 272 *Romulea* genotypes for 8168 loci biallelic SNPs and an overall missing rate of 9%. Loci in rows and individual in columns.

**Figure S2 :**  Plastome map of Romulea florentii, 151 kbp. Fasta sequence archived on ZENODO : DOI 10.5281/zenodo.21415909.
